#  Meconium Peritonitis with Multiple Ileal Perforations in a Twin 

**Published:** 2016-01-01

**Authors:** Samuel Wabada

**Affiliations:** Department of Pediatric Surgery, University of Maiduguri Teaching Hospital, Nigeria

**Dear Sir**

The patient was a 6-hour-old boy, a first twin delivered at the federal medical centre Yola by a 20-year-old house wife per vaginum after 39 weeks of unsupervised pregnancy. The abdomen of the index neonate was immediately noticed to be distended at birth and he did not pass meconium for more than 24 hours. There was an accompanied respiratory difficulty. There was no vomiting. The second twin was doing fine. The child weighted 2.2 kg, and was dyspneic and tachypneic with pulse of 165/min. The abdomen was uniformly distended and shiny. Rectal examination was unremarkable. Plain abdominal x-ray showed pneumoperitoneum with dilated central bowel loops. No calcification was seen. The patient was taken for exploratory laparotomy after resuscitation. The intraoperative findings were meconium ascites, three ileal perforations (Fig. 1) on the antimesenteric border, each not less than 2cms apart, and approximately 20cm from the ileocecal junction. There were fibrinous adhesions involving loops of the distal ileum. There was a bolus of large meconium distal to the perforations with collapsed loops of bowel distal to it. The bowel proximal to the perforations was moderately dilated. This segment of the ileum was resected about 15cm from the ileocecal junction and an ileoileal end-to-end anastomosis was performed. A full thickness rectal biopsy was taken to exclude total colonic aganglionosis. The patient did well, except for developing a superficial surgical site infection which was managed with wound dressing. The histology of the respected ileum showed features of nonspecific inflammation with bile pigments. The rectal biopsy was negative for Hirschsprung’s disease. Breast feeding was introduced on the 5th day after surgery and the patient was discharged on the 10th day. 

**Figure:**
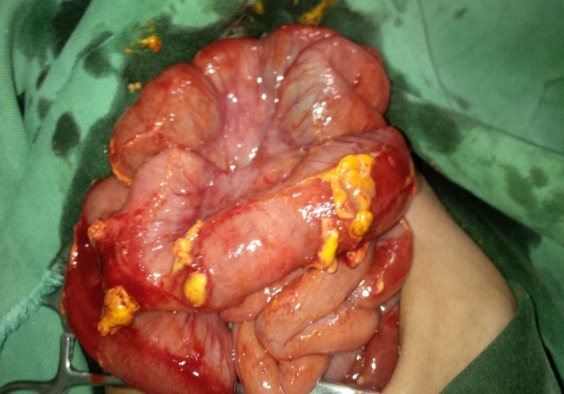
Figure 1: Intraoperative findings showing the ileal perforations.

In utero intestinal perforation could be due to various causes and can lead to generalized, fibro-adhesive or cystic forms of meconium peritonitis 1]. Generalized meconium peritonitis develops if the perforation occurs late in the prenatal period. [2,3]. The diffuse nature of the peritonitis initiates the inflammatory process, and calcification is absent because the inflammatory period is short. The typical presentation of abdominal distention at birth and failure to pass meconium are common in the majority of cases [3,4]. The presence of fever may suggest sepsis because of bacterial colonization in patients with delayed presentation. Delay in presentation increases mortality in these patients [5]. Prenatal diagnosis is vital in finding the predisposing factors of meconium peritonitis [4]. Antenatal diagnostic features include: bowel dilatation, ascites, and polyhydramnios. Calcification is also a vital diagnostic feature, which can easily be seen on a post-natal plain abdominal x-ray, if present [4]. The pathogenesis of calcification in meconium peritonitis has been linked to the precipitation of calcium salts in the meconium, or the inflammatory response of keratin debris in the meconium [5]. In our case, the other twin was normal indicating local factors in the affected fetus played a role in the pathogenesis of meconium disease and maternal systemic factors are less likely involved.

## Footnotes

**Source of Support:** Nil

**Conflict of Interest:** None
